# IL-33 induces innate lymphoid cell–mediated airway inflammation by activating mammalian target of rapamycin

**DOI:** 10.1016/j.jaci.2012.05.018

**Published:** 2012-11

**Authors:** Robert J. Salmond, Ananda S. Mirchandani, Anne-Gaelle Besnard, Calum C. Bain, Neil C. Thomson, Foo Y. Liew

**Affiliations:** a Division of Immunology, Infection and Inflammation, University of Glasgow, Glasgow, United Kingdom; b CEGMR, King Abdulaziz University, Jeddah, Saudi Arabia

**Keywords:** IL-33, T_H_2, innate lymphoid cells, asthma, mammalian target of rapamycin, rapamycin, AHR, Airway hyperresponsiveness, APC, Allophycocyanin, BAL, Bronchoalveolar lavage, ICOS, Inducible costimulator, ILC, Innate lymphoid cell, Lin, Lineage-specific marker, mTOR(C1/2), Mammalian target of rapamycin (complex 1/2), PE, Phycoerythrin, PI3K, Phosphoinositide 3-kinase, rpS6, Ribosomal protein S6, S6K1, Ribosomal protein S6 kinase 1, TCR, T-cell receptor, TSLP, Thymic stromal lymphopoietin, WT, Wild-type

## Abstract

**Background:**

The IL-1 family cytokine IL-33 is involved in the induction of airway inflammation in allergic patients and after viral infection. Several cell types, including CD4^+^ T_H_2 cells and the recently described type 2 innate lymphoid cells (ILCs), are targets for IL-33, yet the mechanisms by which this cytokine modulates their activation are not clear.

**Objectives:**

Our goal was to investigate a role for mammalian target of rapamycin (mTOR) signaling in the activation of T_H_2 and ILC responses and the induction of airway inflammation by IL-33.

**Methods:**

We biochemically determined the effect of IL-33 on mTOR activation in T_H_2 cells and ILCs and examined the effect of this signaling pathway *in vivo* using a murine model of IL-33–induced lung inflammation.

**Results:**

We found that IL-33 induces mTOR activation through p110δ phosphoinositide 3-kinase and that blockade of the mTOR pathway inhibited IL-33–induced IL-5 and IL-13 production by T_H_2 cells and ILCs. Furthermore, use of a ribosomal protein S6 kinase 1 inhibitor implicated a role for ribosomal protein S6 kinase 1 in IL-33–induced mTOR-dependent cytokine production. Intranasal administration of IL-33 to wild-type mice induced airway inflammation, whereas adoptive transfer of wild-type ILCs to IL-33 receptor–deficient *(St2*^*−/−*^*)* mice recapitulated this response. Importantly, coadministration of the mTOR inhibitor rapamycin reduced IL-33–dependent ILC, macrophage, and eosinophil accumulation; cytokine secretion; and mucus deposition in the airways.

**Conclusion:**

These data reveal a hitherto unrecognized role of mTOR signaling in IL-33–driven, ILC-dependent inflammation *in vivo* and suggest that manipulation of this pathway might represent a target for therapeutic intervention for airway inflammation.

The IL-1 family member IL-33 is a pleiotropic cytokine that has been implicated in the induction of airway hyperresponsiveness (AHR) in allergic patients and after viral infection. Administration of IL-33 to mice induces airway inflammation independently of adaptive immune responses,^1^ whereas increased levels of expression of IL-33 in bronchial epithelia are associated with increased severity of disease in asthmatic patients.^2-4^ An understanding of the mechanisms and cellular targets of IL-33 might therefore lead to therapeutic intervention in patients with asthma and allergic inflammation.^5^

IL-33 promotes CD4^+^ T-cell differentiation to an atypical T_H_2 phenotype characterized by the expression of IL-5 and IL-13 but not IL-4.^6^ IL-33 also enhances the differentiation of “alternatively activated” macrophages^7,8^ and stimulates mast cell,^9-12^ basophil,^13,14^ and eosinophil^13,15,16^ responses. Interestingly, a number of novel innate lymphoid cell (ILC) populations that are important for the induction of type 2 responses^17-20^ and tissue remodeling^21^ have recently been described. These cells are of lymphoid origin^22^ and are characterized by their rapid production of IL-5 and IL-13 in response to IL-25 and IL-33.^17,19,20^ Importantly, IL-33–driven type 2 ILCs were recently shown to contribute to AHR after viral infection and in protease-, ovalbumin-, and glycolipid–induced murine models of airway inflammation.^23-28^

Recently, much work has focused on the requirement for the mammalian target of rapamycin (mTOR) signaling pathway in driving immune responses.^29,30^ mTOR is a serine/threonine kinase that links signaling in response to growth factors and nutrients and is important for the regulation of cell growth, metabolism, and differentiation. Interestingly, mTOR activity is important for the induction of AHR by CD4^+^ T_H_2 cells in response to house dust mite allergens.^31^ By contrast, the role of mTOR in IL-33 signaling and in type 2 ILC effector responses is unknown.

Here we describe an important role for mTOR signaling in IL-33–dependent T_H_2 and ILC effector responses both *in vitro* and *in vivo*. IL-33 directly induced the activation of mTOR in a phosphoinositide 3-kinase (PI3K) p110δ–dependent manner. Furthermore, inhibition of mTOR reduced IL-33–driven IL-5 and IL-13 expression by both T_H_2 cells and ILCs *in vitro*. We also show that IL-33–induced airway inflammation was mediated by ILCs and that rapamycin reduced ILC accumulation, macrophage and eosinophil infiltration, cytokine secretion, and mucus deposition in the lung. These data uncover a hitherto unrecognized critical role for mTOR signaling in the biological effects of IL-33 and the effector responses of type 2 ILCs.

## Methods

### Mice

BALB/c mice were from Harlan-Olac (Bicester, United Kingdom). BALB/c *St2*^*−/−*^ mice^32^ were bred and maintained at the University of Glasgow. All experiments were performed in accordance with UK Home Office guidelines.

### Cell lines

D10.G4.1 cells were maintained in complete RPMI 1640 medium (Gibco, Carlsbad, Calif) containing 20 pg/mL IL-1α (R&D Systems, Minneapolis, Minn) and 10% T-Stim culture supplement (BD Biosciences, San Jose, Calif). For biochemical analysis, D10.G4.1 cells were incubated in complete medium in the absence of IL-1α or T-Stim for 24 hours before stimulation. CD4^+^ T cells were purified from lymph nodes of BALB/c or *St2*^*−/−*^ mice by means of negative selection (Miltenyi Biotec, Bergisch Gladbach, Germany) to generate primary T_H_2 cells. Cells were stimulated on anti-CD3ε (BD PharMingen, San Jose, Calif)–coated plates in the presence of anti-CD28 (BD PharMingen), anti–IFN-γ (R&D Systems), and 10 ng/mL recombinant IL-4 and IL-2 (R&D Systems) for 4 days. Cells were restimulated for 2 further 4-day rounds of polarization under similar conditions without IL-2. Before restimulation, cells were incubated overnight in complete RPMI in the absence of cytokines and T-cell receptor (TCR) stimulation.

### Intranasal IL-33 administration, isolation of ILCs, and adoptive cell transfer

Mice were anesthetized with isoflurane and 30 μL of PBS ± 1 μg of IL-33 ± 1 mg/kg rapamycin inoculated into the nasal passage. For ILC isolation, mice were treated for 5 days with IL-33. Lungs were collected on day 6 and digested in 125 μg/mL Liberase TL and 100 μg/mL DNAse 1 (Roche Diagnostics, Mannheim, Germany). Nonadherent cells were stained with ST2–fluorescein isothiocyanate, lineage markers (B220, FcεRI, CD11b, and CD3ε)–phycoerythrin (PE), CD278-PerCP/Cy5.5, CD45-Alexafluor 700, and UVE/DEAD fixable Aqua Dead cell stain (Life Technologies, Carlsbad, Calif) and sorted with a BD FACS Aria. Cells were rested overnight before *in vitro* analyses. For transfer, 10^6^ ILCs in 30 μL of PBS were inoculated intranasally, as described, followed by PBS ± IL-33 ± rapamycin.

### Analysis of bronchoalveolar lavage fluid and lungs

Trachea were cannulated, 800 μL of PBS was flushed into the lungs, and the fluid was collected. Bronchoalveolar lavage (BAL) fluid was centrifuged, and supernatants were collected. Cell pellets were resuspended in PBS and counted. Cells (10^5^) were used for cytospin preparations and stained by using the Romanovsky method (Raymond A Lamb, Eastborne, United Kingdom). Cell morphology was assessed microscopically under oil immersion.

### Cell stimulation

Cells were preincubated in the presence or absence of 100 nmol/L rapamycin (Calbiochem, Nottingham, United Kingdom), 100 nmol/L Torin-1 (Tocris Bioscience, Bristol, United Kingdom), 5 μmol/L IC87114 (a gift from Dr K. Okkenhaug, Babraham Institute, United Kingdom), or 10 μmol/L ribosomal protein S6 kinase 1 (S6K1) inhibitor PF-4708671 (Sigma-Aldrich, St Louis, Mo) for 30 minutes. Inhibitors were used at concentrations previously determined as selective for intended targets.^33-36^ Cells were stimulated with IL-33 (BioLegend, San Diego, Calif) ± IL-2, IL-7, or thymic stromal lymphopoietin (TSLP), 10 ng/mL unless otherwise stated, for the time periods indicated.

Additional information on experimental procedures is included in the Methods section in this article's Online Repository at www.jacionline.org.

## Results

### IL-33 induces mTOR activation through ST2 and PI3K p110δ

**Fig 1:**
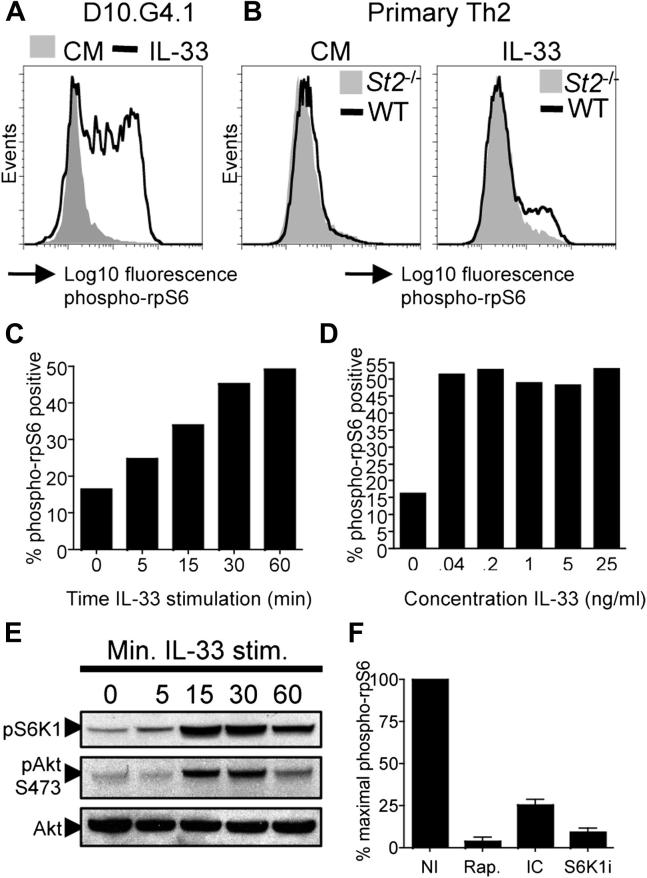
IL-33 induces mTOR activation in T_H_2 cells. **A** and **B,** Histograms show phospho-rpS6 in D10.G4.1 or *WT* and *St2*^*−/−*^ T_H_2 cells. *CM*, Complete medium. **C** and **D,** Time course and dose response of IL-33–induced rpS6 phosphorylation in D10.G4.1 cells. **E,** Western blots of phospho-Akt and S6K1. **F,** Relative levels of phospho-rpS6 in D10.G4.1 cells after stimulation with IL-33 in the presence of no inhibitor *(NI)*, rapamycin (*Rap*.; 100 nmol/L), IC87114 (*IC*; 5 μmol/L), or PF-4708671 (*S6K1i*; 10 μmol/L). *Error bars* represent SDs (n = 4).

We tested the ability of IL-33 to induce phosphorylation of the mTOR target ribosomal protein S6 (rpS6) in the murine T_H_2 cell clone D10.G4.1. Intracellular staining and flow cytometry indicated that IL-33 induced robust phosphorylation of rpS6 (Fig 1, *A*). Importantly, IL-33 also induced rpS6 phosphorylation in primary T_H_2 cells derived from wild-type (WT) but not *St2*^*−/−*^ mice (Fig 1, *B*). Dose-response and kinetic studies indicated that as little as 40 pg/mL IL-33 was sufficient to induce maximal levels of rpS6 phosphorylation that was apparent at 15 minutes and remained high for at least 1 hour after stimulation of D10.G4.1 cells (Fig 1, *C* and *D*). Furthermore, IL-33 induced phosphorylation of the mTOR complex 1 (mTORC1) substrate S6K1 and the mTOR complex 2 (mTORC2) substrate Akt (Fig 1, *E*).

**Fig E1:**
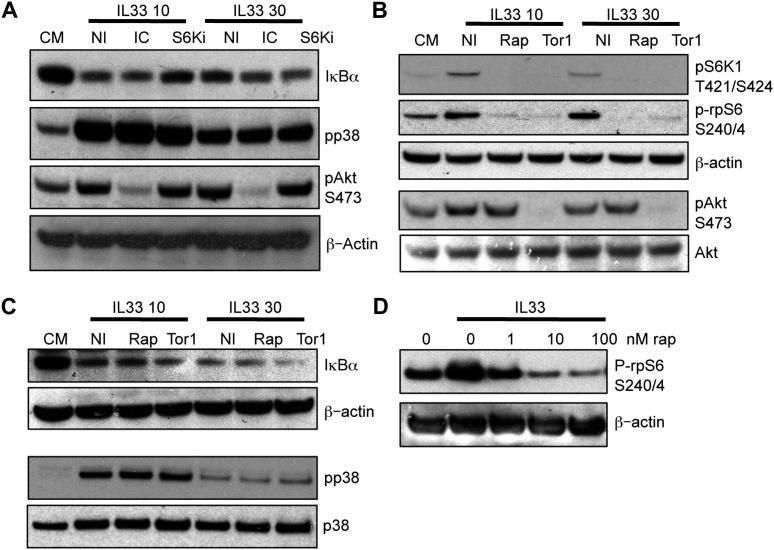
Efficacy and specificity of pharmacologic inhibitors. **A,** Effects of IC87114 (*IC*; 5 μmol/L) and S6K1 inhibitor (*S6K1i*; 10 μmol/L) on IκBα degradation, p38, and Akt phosphorylation. **B** and **C,** Effects of rapamycin (*Rap*; 100 nmol/L) and Torin-1 (*Tor1*; 100 nmol/L) on IL-33–induced S6K1, rpS6, and Akt phosphorylation (Fig E1, *B*) and p38 activation and IκBα degradation (Fig E1, *C*). **D,** Dose-response effects of rapamycin on IL-33–induced rpS6 phosphorylation.

To test the IL-33–induced signaling pathway, resulting in mTOR activation, we used a series of pharmacologic inhibitors. Our previous report has shown that TCR-driven mTOR activation requires the PI3K p110δ isoform.^36^ Furthermore, IL-33 induces activation of PI3K in vascular tissue^37^ and eosinophils.^38^ Pretreatment of D10.G4.1 cells with the p110δ-selective inhibitor IC87114 reduced IL-33–induced rpS6 phosphorylation by 75% (Fig 1, *F*) and markedly inhibited Akt phosphorylation (see Fig E1, *A*, in this article's Online Repository at www.jacionline.org), indicating an upstream role for PI3K p110δ in the activation of both mTORC1 and mTORC2. Rapamycin completely blocked rpS6 phosphorylation (Fig 1, *F*) but did not inhibit Akt phosphorylation (see Fig E1, *B*), confirming the selectivity of the drug for mTORC1 in short-term assays.^39^ By contrast, the mTOR kinase inhibitor Torin-1 completely prevented IL-33–induced rpS6, S6K1, and Akt phosphorylation (see Fig E1, *B*). As expected, an S6K1-specific inhibitor PF-4708671^35^ blocked rpS6 phosphorylation (Fig 1, *F*) but did not affect Akt phosphorylation (see Fig E1, *A*). Importantly, treatment of T_H_2 cells with rapamycin, Torin-1, IC87114, or S6K1 did not affect canonical IL-33–induced nuclear factor κB and p38 mitogen-activated protein kinase signaling pathways, indicating the selectivity of the inhibitors (see Fig E1, *A* and *C*). Together, these data indicate that IL-33 induces mTOR activation and phosphorylation of downstream effector proteins through ST2 signaling and activation of PI3K p110δ.

### mTOR is required for optimal induction of IL-5 and IL-13 production by IL-33

**Fig E2:**
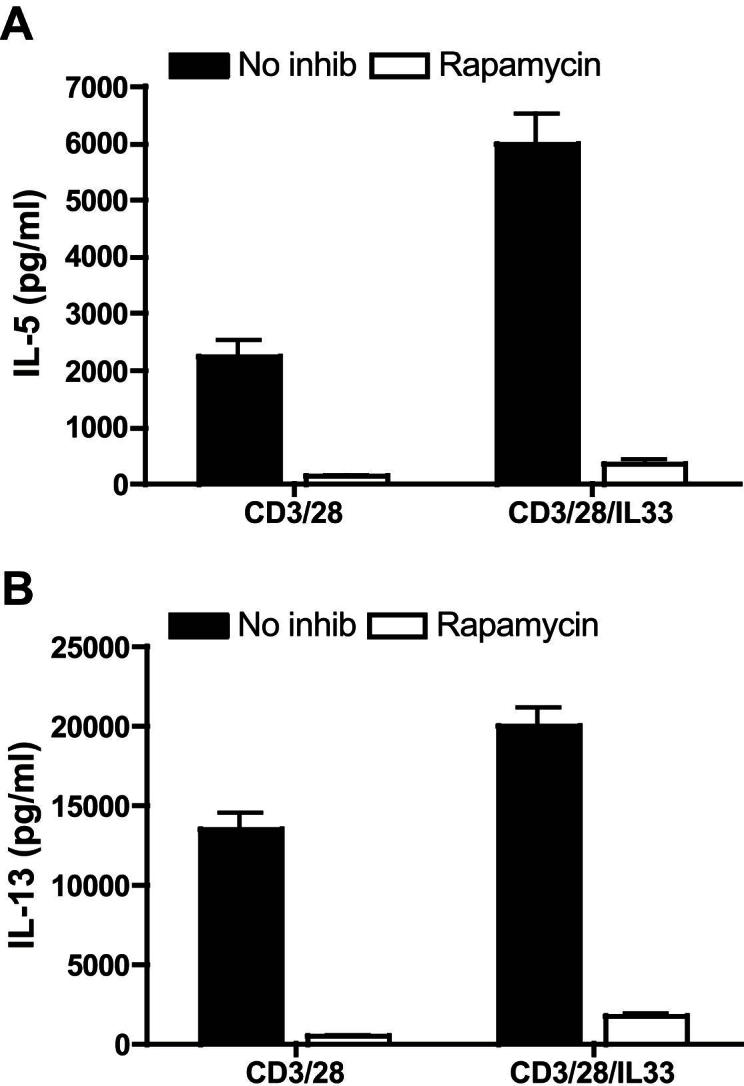
Rapamycin inhibits the effects of IL-33 on TCR-induced differentiation. Naive T cells were stimulated with CD3 and CD28 in the presence or absence of IL-33 and rapamycin (100 nmol/L). Levels of IL-5 **(A)** and IL-13 **(B)** in supernatants were assessed by means of ELISA. *Error bars* represent SEMs (n = 3).

**Fig 2:**
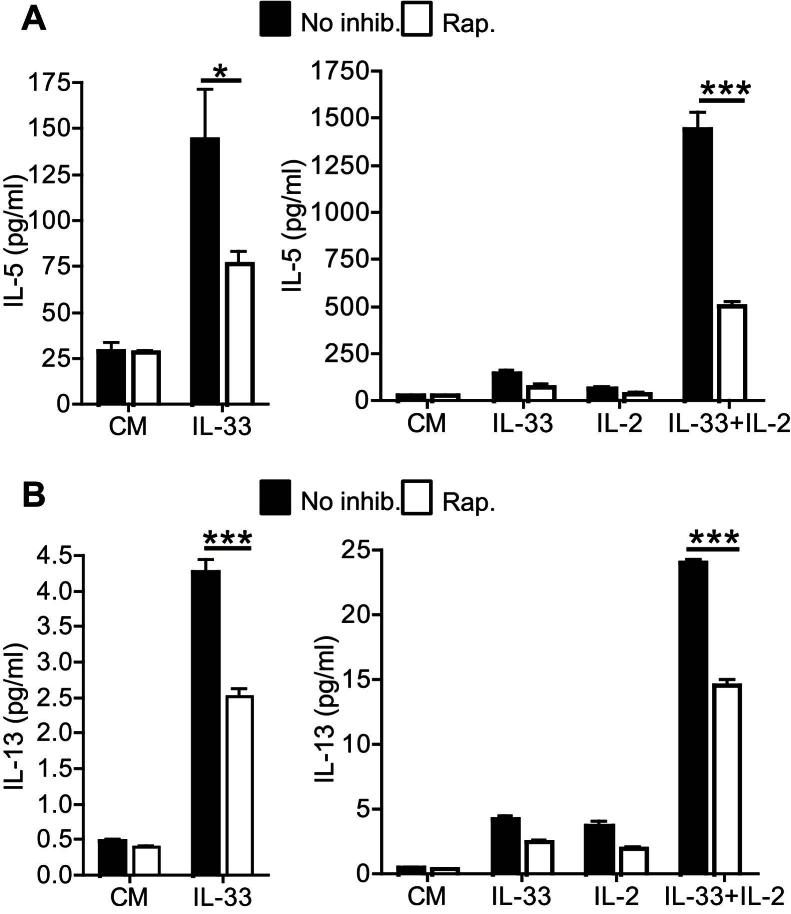
mTOR regulates IL-33–induced cytokine production. Effects of rapamycin (*Rap*.; 100 nmol/L) on levels of IL-5 **(A)** and IL-13 **(B)** induced by IL-33 ± IL-2. *CM*, Complete medium. ∗*P* < .05 and ∗∗∗*P* < .001, unpaired Student *t* test).

A role for mTOR in IL-33–induced T_H_2 cytokine production was assessed. Primary T_H_2 effector cells were restimulated for 24 hours in the absence of TCR stimulation with IL-33 ± IL-2. ELISA analysis indicated that IL-33 or IL-2 alone induced significant levels of IL-5 (Fig 2, *A*) and IL-13 (Fig 2, *B*) production. However, a combination of IL-33 and IL-2 induced strongly increased amounts of IL-5 and IL-13, indicating a synergistic effect (Fig 2). Importantly, treatment of cells with rapamycin inhibited IL-5 production by 70% and IL-13 production by 50% in response to IL-33 ± IL-2 (Fig 2), indicating an important role for mTOR signaling in these processes. We performed experiments to assess the role of mTOR in the effects of IL-33 on TCR-induced polarization of naive T cells. Naive CD4^+^ T cells were stimulated with CD3 and CD28 antibodies ± IL-33 ± rapamycin for 4 days, and levels of cytokines in tissue supernatants were measured. As previously reported,^6^ IL-33 enhanced IL-5 and IL-13 levels (see Fig E2 in this article's Online Repository at www.jacionline.org). Rapamycin treatment profoundly inhibited levels of IL-5 and IL-13 irrespective of whether the cells had been stimulated in the presence of IL-33 (see Fig E2), confirming the vital role for mTOR in differentiation of naive T cells.^40^

**Fig E3:**
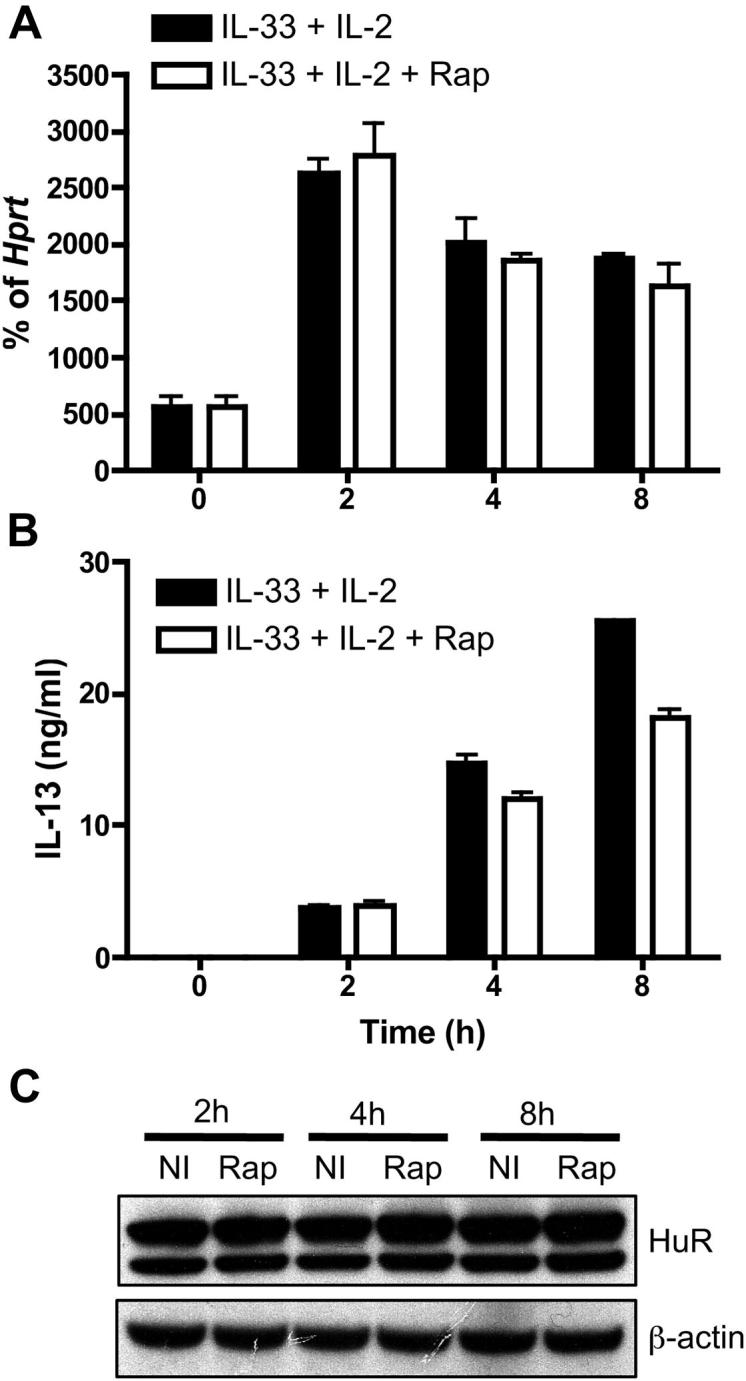
Rapamycin inhibits IL-13 protein production independently of effects on *Il13* mRNA or HuR expression. **A** and **B,** Bar charts show levels of *Il13* mRNA expression in T_H_2 cells (Fig E3, *A*) or protein in supernatants (Fig E3, *B*) after stimulation with IL-33 + IL-2 ± rapamcycin (*Rap*; 100 nmol/L). Data depict values from 1 of 3 repeated experiments, and *error bars* represent SDs (n = 2). **C,** Western blot showing levels of HuR expression in T_H_2 cells stimulated as described above.

To understand the mechanism by which rapamycin inhibited cytokine production, we assessed levels of *Il13* mRNA. Quantitative PCR analysis showed that levels of *Il13* in T_H_2 cells peaked at 2 hours after stimulation with IL-33 + IL-2 (see Fig E3, *A*, in this article's Online Repository at www.jacionline.org). Interestingly, *Il13* mRNA levels were not affected by rapamycin treatment up to at least 8 hours of IL-33 + IL-2 stimulation (see Fig E3, *A*), whereas differences in the levels of IL-13 protein in culture supernatants were already apparent at this time point (see Fig E3, *B*). Furthermore, Western blotting showed that rapamycin did not affect levels of the HuR protein, suggesting that mTOR signals did not affect mRNA stability through this protein (see Fig E3, *C*).

**Fig 3:**
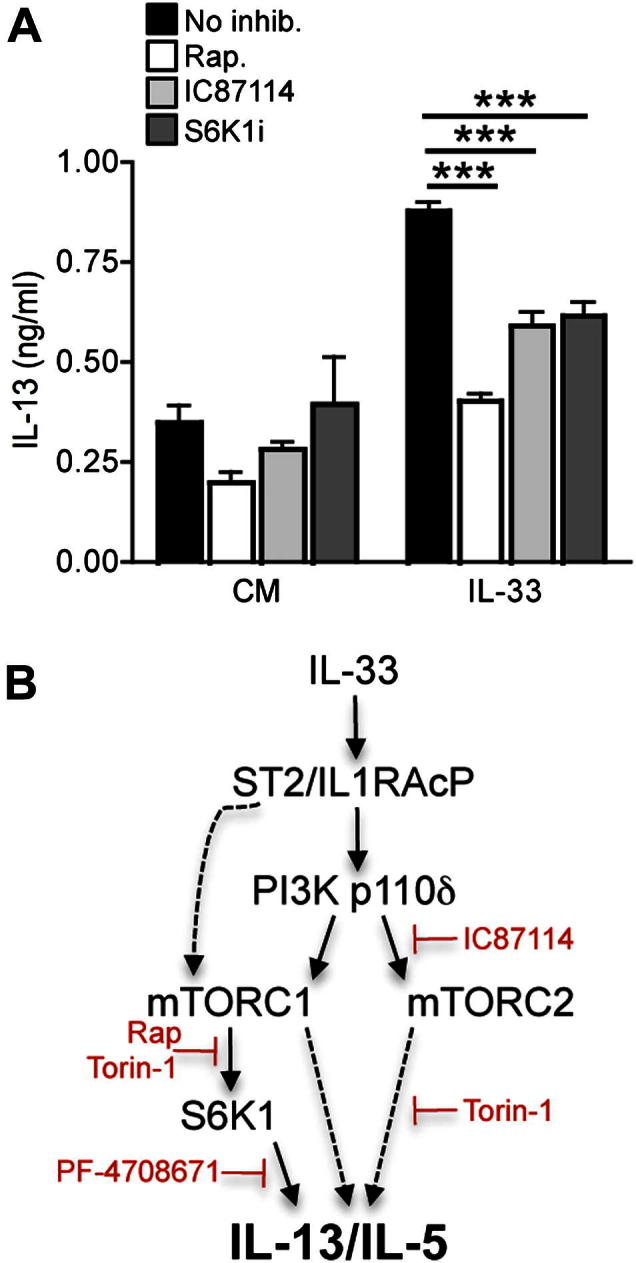
PI3K p110δ and S6K1 inhibitors reduce IL-33–induced IL-13 production. **A,** Comparison of the effects of rapamycin (*Rap*.; 100 nmol/L), IC87114 (5 μmol/L), and PF-4708671 (*S6K1i*; 10 μmol/L) on levels of IL-33–induced IL-13 produced by T_H_2 cells. *Error bars* represent SDs (n = 3-12). Data represent one of 3 repeated experiments. *CM*, Complete medium. ∗∗∗*P <* .001, unpaired Student *t* test. **B,** Schematic representation of the role of the mTOR pathway in IL-33–induced cytokine production.

Finally, we tested the role of the upstream activator of mTOR, PI3K p110δ, and the mTORC1 effector S6K1 in IL-33–induced cytokine production. Treatment of T_H_2 cells with the p110δ inhibitor IC87114 partially inhibited IL-33–induced IL-13 production (Fig 3, *A*), confirming a role for p110δ in IL-33–driven cytokine production. Similarly, incubation of cells with the S6K1 inhibitor PF-4708671 reduced levels of IL-33–induced IL-13 production by approximately 40% (Fig 3, *A*). However, IC87114 and PF-4708671 were less potent suppressors of IL-13 production than rapamycin. Taken together, these data indicate that mTOR activation is important for IL-33–induced cytokine production and suggest that this process is, in part, mediated by mTORC1-induced activation of S6K1 (Fig 3, *B*).

### Rapamycin reduces IL-33–induced airway inflammation and cytokine production *in vivo*

**Fig 4:**
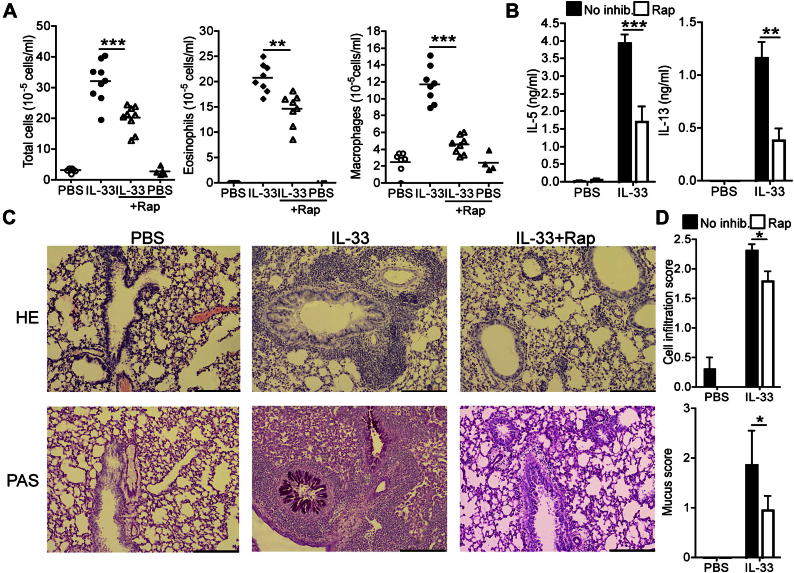
Rapamycin reduces IL-33–induced airway inflammation. **A,** Effect of rapamycin (*Rap*; 1 mg/kg) on IL-33–induced total BAL cell, eosinophil, and macrophage accumulation. Values are from individual mice, and *bars* represent means. **B,** Levels of IL-5 and IL-13 in BAL fluid. **C,** Histologic analysis of lung sections after hematoxylin and eosin *(HE)* or periodic acid–Schiff *(PAS)* staining. *Scale bars* = 200 μm. Data are representative of 3 to 8 mice per group. **D,** Histologic scores of cell infiltration and mucus deposition. *Error bars* represent SEMs. ∗*P* < .05, ∗∗*P* < .01, and ∗∗∗*P* < 0.001, unpaired Student *t* test.

Having determined an important role for mTOR in the induction of T_H_2 cell cytokine production, we sought to examine the function of this signaling pathway in the induction of airway inflammation by IL-33 *in vivo*. We assessed the effects of intranasal administration of recombinant IL-33 in the presence or absence of rapamycin on 5 consecutive days to BALB/c mice. Analysis of BAL fluid indicated that intranasal IL-33 induced the accumulation of eosinophils and macrophages in the lungs (Fig 4, *A*). Importantly, coadministration of rapamycin strongly reduced IL-33–induced eosinophil and, particularly, macrophage infiltration (Fig 4, *A*). Analysis of BAL fluid showed that IL-33 induced high levels of IL-5 and IL-13 in the airways (Fig 4, *B*). Coadministration of rapamycin strongly inhibited IL-33–induced cytokine production, confirming a requirement for mTOR in this process *in vivo* (Fig 4, *B*).

Histologic analysis confirmed substantial cell infiltration into the lungs of IL-33–treated mice (Fig 4, *C*). Furthermore, periodic acid–Schiff staining of lung sections indicated large amounts of mucus deposition after IL-33 administration (Fig 4, *C*). By contrast, levels of cell infiltration and mucus deposition were reduced in mice coadministered IL-33 and rapamycin (Fig 4, *C* and *D*). Taken together, these data show a potent suppressive effect of the mTOR inhibitor rapamycin on the induction of airway inflammation by IL-33.

### Type 2 ILCs are responsible for IL-33–induced cytokine production during airway inflammation *in vivo*

**Fig E4:**
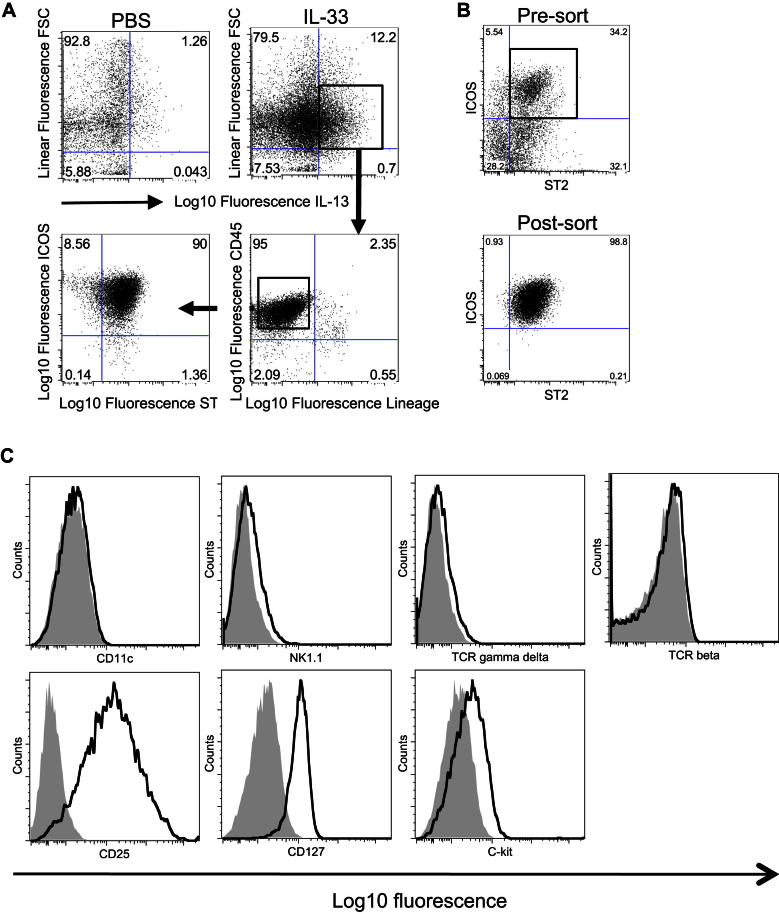
Phenotypic analysis of type 2 ILCs. **A,** Fluorescence-activated cell sorting analysis demonstrating IL-33–elicited IL-13–producing cells present in lung digests are predominantly Lin^−^CD45^+^ICOS^+^ST2^+^ ILCs. **B,** Dot plots depict proportions of gated CD45^+^Lin^−^ type 2 ILCs before and after cell sorting, as assessed by analysis of ST2 and ICOS expression. **C,** Histograms showing cell-surface expression of CD11c, NK1.1, TCRγδ, TCRβ, CD25, CD127, and c-Kit on purified type 2 ILCs. *Shaded histograms* represent isotype controls.

**Fig 5:**
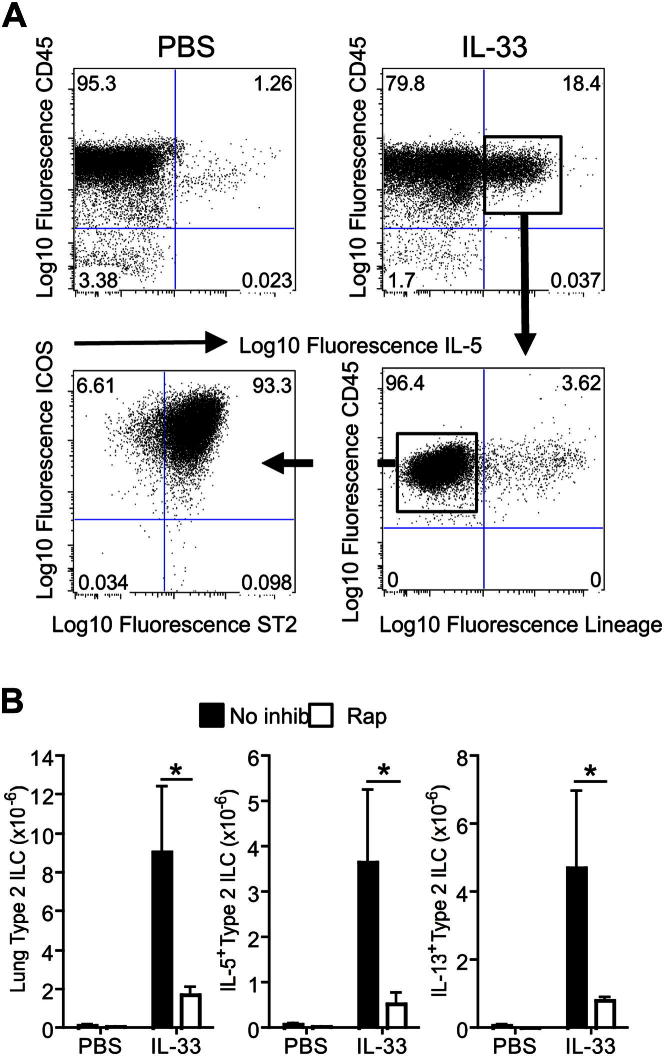
Type 2 ILCs are the predominant cytokine-producing population induced by IL-33 in murine lungs, whereas rapamycin *(Rap)* reduces their accumulation. **A,** Fluorescence-activated cell sorting analysis of IL-5–producing cells present in lung digests. **B,** Effects of rapamycin on total ILCs, IL-5–producing ILCs, and IL-13–producing ILCs. ∗*P* < .05, unpaired Student *t* test. *Error bars* represent SDs (n = 3-4 per group), and data represent one of 3 repeated experiments.

To determine which cell type was responsible for IL-33–induced cytokine production in the lungs, we performed multiparameter flow cytometry on cells from mice treated intranasally with IL-33. Cells from lung digests were restimulated with phorbol ester and ionomycin and stained for intracellular IL-5 (Fig 5, *A*) or IL-13 (see Fig E4, *A*, in this article's Online Repository at www.jacionline.org). Cells were further analyzed for surface expression of lineage-specific markers (Lin), including B220, CD11b, FcεRI, and CD3ε. The majority of cytokine-positive cells were CD45^+^Lin^−^ (Fig 5, *A*). Further analysis of CD45^+^Lin^−^ cells from the lungs of IL-33–treated mice indicated that these cells did not express CD11c, NK1.1, TCRαβ, or TCRγδ but did express high levels of inducible costimulator (ICOS), ST2, CD25, and CD127 and low levels of c-Kit (Fig 5, *A*, see Fig E4), which is consistent with the phenotype of ILC populations described recently by several independent groups.^19,21^ Furthermore, analysis of cell numbers showed that intranasal administration of IL-33 potently induced the accumulation of CD45^+^Lin^−^ST2^+^ICOS^+^ ILCs in the murine lung (Fig 5, *B*). Interestingly, coadministration of rapamycin dramatically reduced ILC accumulation. Together these data show that IL-33 promotes the accumulation of IL-5– and IL-13–producing type 2 ILCs in the murine lung and that this process is regulated by the mTOR signaling pathway.

### Rapamycin directly inhibits IL-33–induced ILC effector function

**Fig 6:**
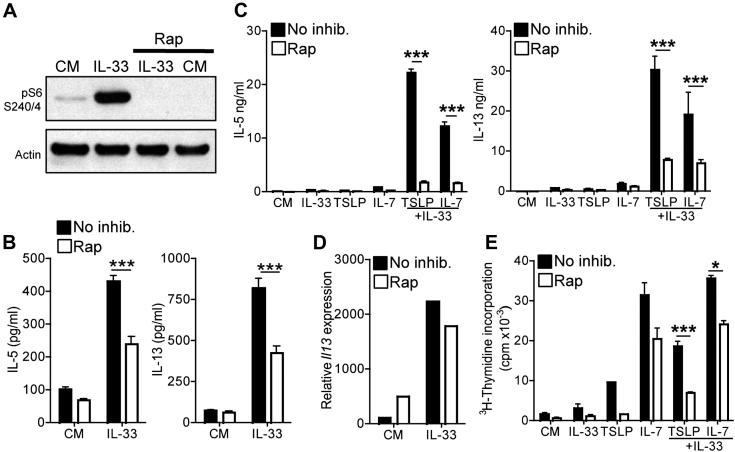
Rapamycin *(Rap)* directly inhibits ILC effector function. **A,** Western blots for phospho-rpS6 in ILCs. Data represent one of 4 experiments. **B** and **C,** Effect of rapamycin (100 nmol/L) on IL-5 and IL-13 induced by IL-33 (Fig 6, *B*) or IL-33 ± TSLP or IL-7 (Fig 6, *C*). **D,** Effect of rapamycin on IL-33–induced *Il13* mRNA levels. Data represent one of 3 experiments. **E,** Effect of rapamycin on ILC proliferation induced by IL-33 ± TSLP or IL-7. All *error bars* represent SDs (n = 3), and all data represent one of at least 3 repeated experiments. *CM*, Complete medium. ∗ *P* < .05 and ∗∗∗*P* < .001, Student *t* test.

Because rapamycin affected ILC accumulation in the lungs (Fig 5, *B*), it was possible that the reduced levels of IL-33–induced cytokines in BAL fluid (Fig 4, *B*) simply represented reduced ILC cell numbers. To directly determine whether mTOR was important for IL-33–driven ILC effector function, we sorted ILCs to greater than 98% purity (see Fig E4, *B*) and tested the effects of rapamycin *in vitro*. Western blot analysis confirmed that IL-33 induced rpS6 phosphorylation in an mTOR-dependent manner in ILCs (Fig 6, *A*). Furthermore, IL-33 alone was sufficient to induce production of IL-5 and IL-13 by ILCs *in vitro* (Fig 6, *B*), whereas combinations of IL-33 with either of the innate cytokines TSLP or IL-7 were strongly synergistic (Fig 6, *C*). Importantly, rapamycin potently reduced levels of IL-5 and IL-13 produced by ILCs in response to IL-33 alone or in combination with TSLP or IL-7 (Fig 6, *B* and *C*). However, rapamycin treatment did not affect basal or IL-33–induced *Il13* mRNA levels (Fig 6, *D*).

In further experiments the effects of IL-33, IL-7, and TSLP on ILC proliferation were assessed. IL-33 alone induced very low levels of proliferation in ILC cultures that were not significantly affected by rapamycin (Fig 6, *E*). By contrast, TSLP and particularly IL-7 potently stimulated ILC proliferation, whereas rapamycin had a modest inhibitory effect (Fig 6, *E*). Taken together, these data confirm an important and direct role for mTOR signaling in IL-33–induced ILC cytokine production.

### Type 2 ILCs are sufficient to mediate IL-33–induced airway inflammation

**Fig E5:**
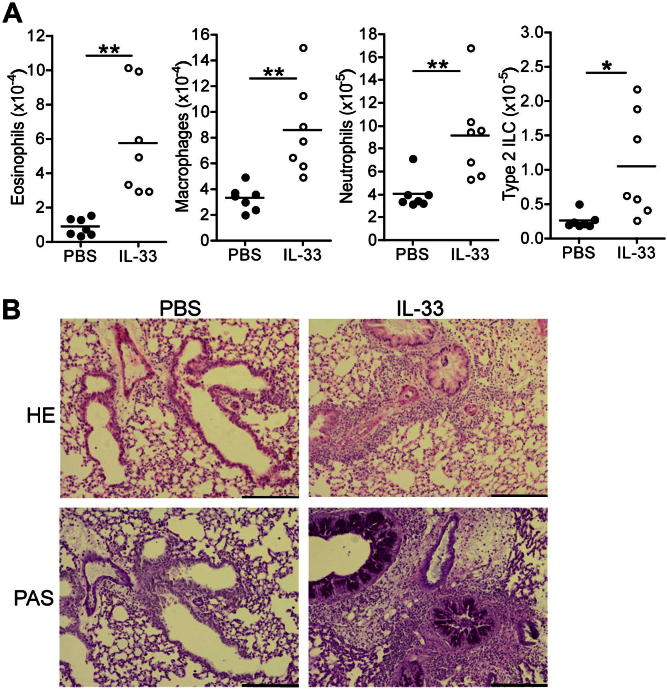
Transferred WT ILCs facilitate IL-33–induced inflammation in *St2*^*−/−*^ recipients. **A,** Eosinophil, macrophage, neutrophil, and donor ILC numbers in BAL fluid and lung digests from IL-33–treated *St2*^*−/−*^ mice receiving WT ILCs. *Horizontal bars* represent means, and data represent individual animals from 2 pooled experiments. **B,** Histologic analysis of lung sections stained with hematoxylin and eosin *(HE)* and periodic acid–Schiff *(PAS)*. Pictures depict representative fields from 1 of 7 to 8 mice per group. *Scale bars* = 200 μm. ∗*P* < .05 and ∗∗*P* < .01.

**Fig 7:**
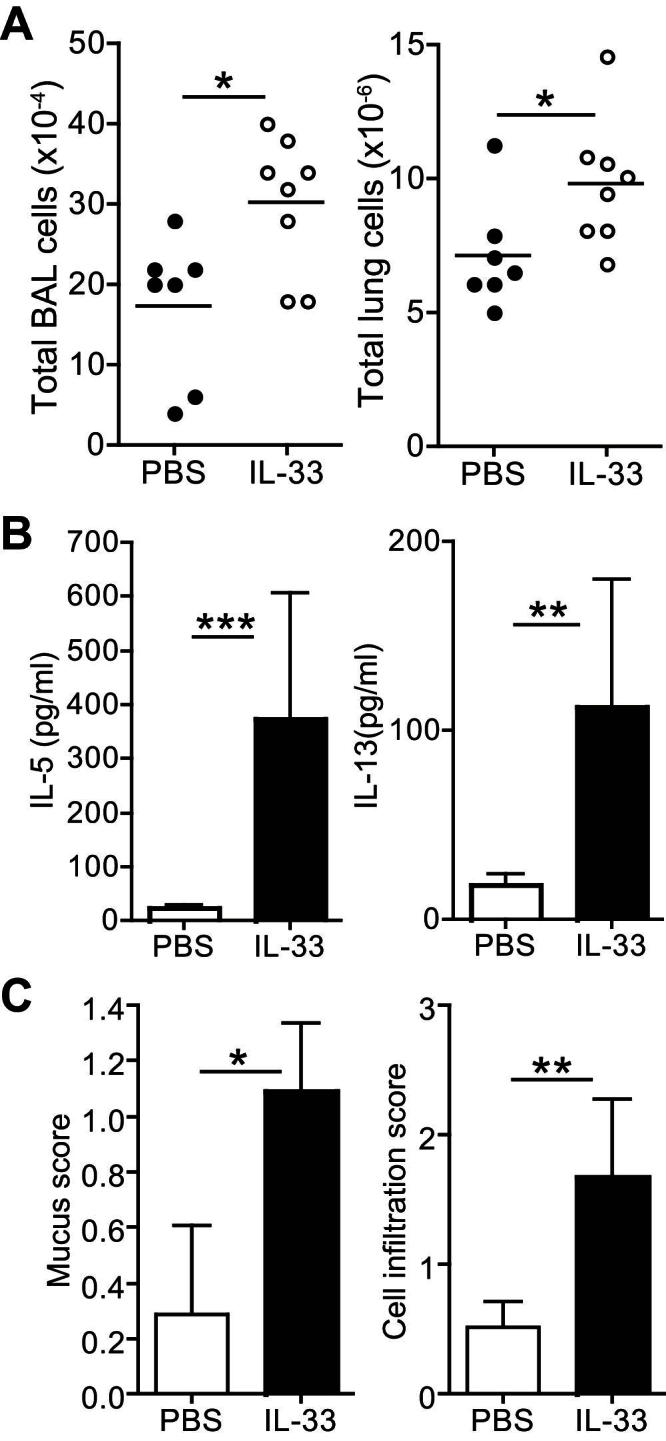
ILCs are sufficient to mediate IL-33–induced airway inflammation. **A,** Cell counts in BAL fluid and lung digests from IL-33–treated *St2*^*−/−*^ mice receiving WT ILCs. **B,** Levels of IL-5 and IL-13 in BAL fluid. Values represent means ± SDs of 3 to 4 mice. **C,** Histologic scores of lung sections. *Error bars* represent SEMs (n = 7-8). ∗*P* < .05, ∗∗*P* < .01, and ∗∗∗*P* < .001.

To determine whether ILCs were sufficient to induce airway inflammation, we developed an adoptive cell transfer system in which ST2-sufficient ILCs were transferred to ST2-deficient hosts. Because ST2 is indispensable for responses to IL-33,^41^ this approach allowed us to investigate the role of ILCs in airway inflammation without potential complications of direct effects of IL-33 on other lung cell populations. Groups of *St2*^*−/−*^ mice were inoculated intranasally with 10^6^ ILCs together with IL-33 or PBS. *St2*^*−/−*^ mice receiving ILCs alone did not show signs of airway inflammation. However, on administration of IL-33, total cell numbers in BAL fluid and lung digests were substantially increased (Fig 7, *A*). Fluorescence-activated cell sorting analysis of lung digests showed that numbers of *St2*^*−/−*^ host eosinophils, macrophages, and neutrophils were increased after intranasal IL-33 administration (see Fig E5, *A*, in this article's Online Repository at www.jacionline.org). Furthermore, the numbers of donor WT ILCs recovered from IL-33–treated mice were markedly higher than those from control mice (see Fig E5, *A*). Levels of IL-5 and IL-13 in the BAL fluid from IL-33–treated mice were also strongly increased, confirming a role for type 2 ILCs in IL-33–induced cytokine production *in vivo* (Fig 7, *B*). Hematoxylin and eosin and periodic acid–Schiff staining of lung tissues from recipient mice indicated that donor WT ILCs were sufficient to induce high levels of cellular infiltration and mucus deposition after administration of IL-33 (Fig 7, *C*, and see Fig E5, *B*). Together, these data show that in the absence of additional IL-33–responsive cells, ILCs are sufficient to induce leukocyte infiltration, cytokine production, and mucus deposition in the lungs of mice in response to IL-33.

**Fig 8:**
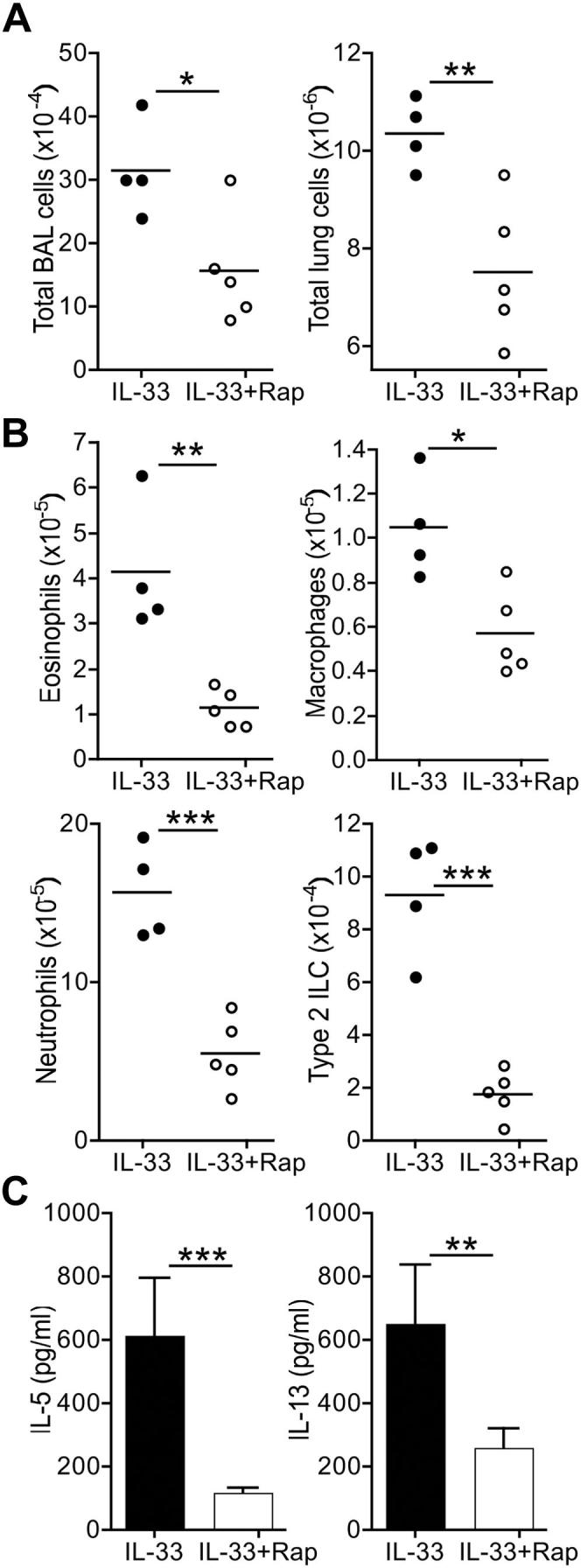
Rapamycin *(Rap)* inhibits ILC-induced airway inflammation. **A,** Cell numbers in BAL fluid and lung digests. *Horizontal bars* represent means, and data represent values from individual mice (4-5 per group) from one of 3 repeated experiments. **B,** Eosinophil, macrophage, neutrophil, and donor ILC cell numbers in lung digests. **C,** Levels of IL-33–induced, ILC-dependent IL-5 and IL-13 in BAL fluid. *Error bars* represent SDs (n = 4-5). ∗*P* < .05, ∗∗*P* < .01, and ∗∗∗*P* < .001, all unpaired Student *t* test.

Finally, experiments were performed to directly test the role of mTOR in IL-33–driven ILC responses *in vivo*. WT ILCs were transferred into *St2*^*−/−*^ mice, and the recipients were then administered intranasal IL-33 ± rapamycin for 5 consecutive days. Rapamycin reduced IL-33–induced ILC-dependent cellular accumulation in the lungs of recipient mice (Fig 8, *A*). Rapamycin reduced eosinophil, macrophage, and neutrophil cell numbers in the lung tissues of recipient mice (Fig 8, *B*), whereas levels of IL-5 and IL-13 in BAL fluid were also substantially lower (Fig 8, *C*). Therefore these data demonstrate a direct regulatory role for mTOR in IL-33–induced type 2 ILC effector function *in vivo*.

## Discussion

Data presented here provide direct evidence that activation of the mTOR signaling pathway by IL-33 is important for stimulating the effector functions of both CD4^+^ T_H_2 cells and type 2 ILCs. Our results have also identified ILCs as critical for the induction of airway inflammation by IL-33 and a requirement for mTOR in this process.

Results from several groups identified novel innate immune cells involved in type 2 immune responses. Moro et al^18^ first described an adipose-associated innate population in the murine mesentery termed “natural helper cells.” This population was characterized by production of IL-5, IL-6, and IL-13 and surface expression of c-Kit, Sca-1, CD127, and ST2. The McKenzie and Locksley laboratories described a similar population that expanded in response to IL-25 and IL-33, produced large amounts of IL-13, and was important for immunity to helminths.^17,19^ Subsequently, the Umetsu and Stockinger laboratories reported an important role for ILC populations in driving airway inflammation in mice.^23,42^ In contrast, Monticelli et al^21^ have reported that a lung ILC population is important for airway remodeling and maintenance of epithelial integrity and lung function after viral infection. Although the precise relationship between these cell populations is not clear, these data have shown that phenotypically similar populations of ILCs are important in the induction of protective type 2 immune responses in parasitic infections and as mediators of both tissue homeostasis and pathologic inflammation in the lung.

In the present study we show that IL-33 directly expands the lung ILC population, which mediates airway inflammation. Importantly, we showed that ILCs were the predominant IL-5– and IL-13–producing cells in the lungs of IL-33–treated mice. Thus WT ILCs were sufficient to mediate IL-33–induced airway inflammation and induce granulocyte, eosinophil, and macrophage infiltration and cytokine production on transfer to *St2*^*−/−*^ mice.

It has become clear that IL-33 is critical for the activation of type 2 ILCs. However, the signaling pathways induced by this cytokine that are responsible for eliciting ILC effector responses have not previously been identified. Our finding that IL-33 induced mTOR activation and that rapamycin potently inhibited IL-33–induced ILC accumulation in the lungs, cytokine production, and airway inflammation demonstrate a vital role for this signaling pathway. We showed that mTOR was activated in response to IL-33 in both T_H_2 cells and ILCs through a pathway that involved PI3K p110δ. We also showed that an S6K1 inhibitor reduced IL-33–induced IL-13 production. Although activation of S6K1 has long been linked to the induction of T-cell proliferation,^43^ little is known of the physiologic role of S6K1 in immune responses. Our data suggest that mTOR regulates IL-33–dependent cytokine production at least in part through the activation of S6K1. Our studies also indicate that inhibition of mTOR affects IL-33–induced cytokine production independently of effects on mRNA levels in both T_H_2 cells and ILCs. These data are consistent with the well-documented role of mTORC1 in the translational regulation of gene expression. Thus S6K1 and additional mTOR targets, including the 4E-binding proteins, are known to regulate the rates of translation initiation.^44^

Recent data have identified human IL-33–responsive ILC populations that are present in increased numbers in nasal polyps of patients with chronic rhinosinusitis.^20^ Furthermore, increased IL-33 expression in bronchial biopsy specimens has been suggested as a clinical marker of severe asthma.^2,3^ Moreover, rapamycin was effective at inhibiting T-cell responses from glucocorticoid-refractory asthmatic patients,^45^ whereas mTOR activity is required for mast cell cytokine production and cell survival.^46^ Therefore our finding that rapamycin potently suppressed IL-33–induced ILC-mediated lung inflammation advances the field considerably and, furthermore, suggests that manipulation of the mTOR pathway might prove beneficial in airway inflammation in human disease.

**Key messages**

• This study identifies an important role for the mTOR signaling pathway in the biological responses of CD4^+^ T_H_2 cells and ILCs elicited by IL-33.
• Abrogation of mTOR signaling with rapamycin reduces T_H_2 cell and ILC cytokine production *in vitro* and alleviates IL-33–induced airway inflammation *in vivo* by reducing ILC accumulation, cytokine secretion, eosinophilia, and mucus deposition in the airways.
• These studies provide a molecular mechanism and identify ILCs as sufficient for the induction of airway inflammation by IL-33.

## Methods

### Flow cytometry

For phospho-rpS6 analysis, cells were fixed in 2% formaldehyde, washed in PBS, permeabilized, and stored in 90% methanol until analysis. For cytokine staining, lung cells were fixed and permeabilized in Cytofix/Cytoperm buffer and washed in Perm/wash buffer (both from BD Biosciences). The following antibodies were used: B220-PE, CD11b-PE, CD117–allophycocyanin (APC), CD25-PE, TCRβ-Biotin, IL-5–APC (all from BD Biosciences), CD4-PE/Cy7, CD11c-APC, NK1.1-PE, CD127-APC, TCRγδ-APC, FcεRIα-PE, CD45–AlexaFluor 700, CD3ε-PE, IL-13–AlexaFluor 647 (all from eBioscience, San Diego, Calif), CD278-PerCP/Cy5.5 (BioLegend), rpS6 Ser240/4-AlexaFluor 488 (Cell Signaling Technology, Danvers, Mass), and ST2–fluorescein isothiocyanate (MD Biosciences, St Paul, Minn). Data were acquired by using FACSCalibur, FACSAria, or LSRII flow cytometers (all from BD Biosciences) and analyzed with FlowJo software (TreeStar, Ashland, Ore).

### Western blotting

Lysates were prepared in RIPA buffer and NuPage sample buffer (Invitrogen, Carlsbad, Calif). Cell equivalents (2.5 Χ 10^5^) were loaded on NuPage minigels (Invitrogen), and proteins were separated by means of electrophoresis and transferred to nitrocellulose by using the I-Blot dry transfer system (Invitrogen). Membranes were blocked in 5% BSA before blotting with phospho-S6K1 Ser421/Thr424, phospho-Akt Ser473, phospho-rpS6 Ser240/4, Akt (all from Cell Signaling Technology), and tubulin (Santa Cruz Biotechnology, Santa Cruz, Calif) antibodies. Horseradish peroxidase–conjugated secondary antibodies were from Cell Signaling. Membranes were incubated with chemiluminescent substrate (Amersham, Piscataway, NJ) and exposed to Medical X-Ray film (Kodak, Rochester, NY).

### Cytokine ELISA

T_H_2 cells (5 × 10^4^ cells) or ILCs (1 × 10^5^ cells) were cultured in triplicate wells, as indicated, for 24 hours. Cytokines in culture supernatants or BAL fluid were quantified by means of ELISA with IL-5 capture and biotinylated detection (both from BD Biosciences) or murine IL-13-Ready-SET-Go! (eBioscience).

### RNA isolation and quantitative PCR

T_H_2 cells (10^6^) or ILCs were preincubated in the presence or absence of 50 nmol/L rapamycin before stimulation with IL-33 ± IL-2 for 4 hours. RNA was prepared by using RNeasy mini-kits (Qiagen, Hilden, Germany), and cDNA was generated with MultiScribe reverse transcriptase (Applied Biosystems, Foster City, Calif). TaqMan probes, primer sets, and an ABI Prism 7900 Sequence Detection System instrument real-time PCR system (Applied Biosystems) were used for real-time quantitative PCR amplification of cDNA. Levels of *Il13* were normalized to those of *Hprt*.

### Cell proliferation

T_H_2 cells or ILCs (5 × 10^4^ cells) were cultured in the presence of IL-33 ± IL-2, IL-7, or TSLP (all 10 ng/mL) ± rapamycin (50 nmol/L). Cells were pulsed with 0.037 MBq of tritiated thymidine in the last 16 hours of a 72-hour culture.
